# Effect of canagliflozin on insulin sensitivity in insulin-dysregulated horses as assessed by euglycemic hyperinsulinemic clamp: a randomized, placebo-controlled trial

**DOI:** 10.1093/jvimsj/aalag285

**Published:** 2026-10-29

**Authors:** Elin Svonni, Peter Michanek, Peter Bergsten, Sanna Lindåse, Johan Bröjer

**Affiliations:** Department of Clinical Sciences, Swedish University of Agricultural Sciences, 750 07 Uppsala, Sweden; Department of Animal Biosciences, Swedish University of Agricultural Sciences, 750 07 Uppsala, Sweden; Department of Women’s and Children’s Health; Centre for Health and Sustainability, Uppsala University, 751 85 Uppsala, Sweden; Department of Clinical Sciences, Swedish University of Agricultural Sciences, 750 07 Uppsala, Sweden; Department of Clinical Sciences, Swedish University of Agricultural Sciences, 750 07 Uppsala, Sweden

**Keywords:** equine, equine metabolic syndrome, hyperinsulinemia, insulin dysregulation, sodium-glucose co-transporter 2 inhibitor, SGLT2 inhibitor

## Abstract

**Background:**

Insulin resistance, enhanced β-cell responsiveness, and lower insulin clearance contribute to hyperinsulinemia in horses with insulin dysregulation. Canagliflozin (CAN) reduces hyperinsulinemia in horses, but its effect on insulin sensitivity (IS) remains unclear.

**Hypothesis/Objectives:**

Evaluate the effects of CAN on IS and insulin clearance using a euglycemic hyperinsulinemic clamp (EHC) and on β-cell responsiveness using a graded glucose infusion test (GGI) in insulin-dysregulated (ID) horses.

**Animals:**

Twenty privately owned ID mares.

**Methods:**

In this randomized, double-blind, placebo-controlled study, horses received CAN (0.4 mg/kg, *n* = 10) or placebo (PBO, *n* = 10) PO once daily for 4 weeks. Insulin sensitivity and insulin metabolic clearance rate (MCR) were assessed by EHC, and β-cell responsiveness by GGI.

**Results:**

Insulin sensitivity (M/I) was lower with CAN than PBO (1.5 ± 0.2 vs 2.7 ± 0.2 [mg/kg/min × 10^3^]/[μIU/mL]; LS mean difference,—1.3 [95% CI, -1.9 to -0.6]; *P* < .001), whereas insulin MCR was higher (6.5 ± 0.3 vs 5.4 ± 0.3 mL/kg/min; LS mean difference, 1.0 [95% CI, 0.3-1.8]; *P* = .01). Beta-cell responsiveness (ΔI vs ΔG) was 43.3% lower with CAN (20.8 [95% CI, 16.9-25.5] vs 36.7 [29.9-45.1] mIU/mmol; ratio of geometric LS means, 0.57 [95% CI, 0.42-0.76]; *P* < .001).

**Conclusions and clinical importance:**

Beta-cell responsiveness was lower and insulin clearance higher in CAN-treated ID mares, despite apparently lower IS. Higher endogenous glucose production in CAN-treated mares might have contributed to an underestimation of IS.

## Introduction

Hyperinsulinemia is the primary concern in insulin-dysregulated (ID) horses, as it can lead to laminitis.^1^^,^^2^ Sodium-glucose co-transporter 2 (SGLT2) inhibitors, including canagliflozin (CAN), have gained increasing attention as a treatment option for ID horses.^3^ These drugs cause higher urinary glucose excretion (UGE) by inhibiting SGLT2 in the proximal renal tubules. In ID horses, which typically exhibit normal or only mildly elevated blood glucose concentrations, the primary therapeutic benefit of SGLT2 inhibitor treatment is a reduction in insulin concentrations.^4–6^

Several factors contribute to hyperinsulinemia in ID horses, including insulin resistance (IR), higher intestinal incretin activity, and lower insulin clearance.^7–9^ Limited evidence also suggests that ID horses might exhibit enhanced β-cell responsiveness, defined as the amount of insulin secreted in response to a given glucose stimulus.^9^ A similar pattern, characterized by enhanced β-cell responsiveness in the presence of IR, is observed in humans during the prediabetic stage and early stages of type 2 diabetes (T2D).^10–12^

Treatment with CAN reduces β-cell responsiveness to both PO and IV glucose challenges in ID horses.^4^^,^^13^ However, the underlying mechanism is not well understood. A hyperbolic relationship between β-cell responsiveness and insulin sensitivity (IS) is demonstrated in horses and other species.^9^^,^^14–16^ This relationship implies that higher IS is accompanied by lower glucose-stimulated insulin secretion (GSIS), provided that the disposition index, and therefore glucose tolerance, remains constant. Thus, improved IS might partly explain the lower β-cell responsiveness observed in CAN-treated ID horses. However, data on the effects of SGLT2 inhibitors on IS in horses are limited. One study found no effect of the SGLT2 inhibitor velagliflozin on IS in ponies with insulin dysregulation, as measured by a combined glucose-insulin tolerance test.^17^ Studies in other species report conflicting results; however, several show improved IS with SGLT2 inhibitor treatment, potentially attributable to reduced glucotoxicity.^18–20^

The primary objective of our study was therefore to determine whether short-term CAN treatment (0.4 mg/kg) affects IS in ID horses evaluated using a euglycemic hyperinsulinemic clamp (EHC), the gold standard for measuring IS. The secondary objectives included evaluation of the effects of CAN on insulin clearance and β-cell responsiveness to IV glucose, assessed using EHC and a graded glucose infusion test (GGI), respectively.

## Materials and methods

A full description of the materials and methods is provided in the Appendix.

### Study design

This randomized, double-blind, parallel-group, single-center, placebo-controlled study evaluated CAN (0.4 mg/kg) vs placebo (PBO), administered PO once daily for 4 weeks, in privately owned ID horses with insulin concentrations >100 μIU/mL during oral sugar test (OST). Experiments were conducted between October 2023 and April 2025.

### Enrollment criteria

Privately owned ID mares were eligible for enrollment. Exclusion criteria included male sex (as urinary catheter placement without sedation is anatomically difficult), age <2 years, pregnancy or lactation, ongoing laminitis, systemic disease, or current medication. A flow diagram of enrollment is shown in Figure 1.

**Figure 1:**
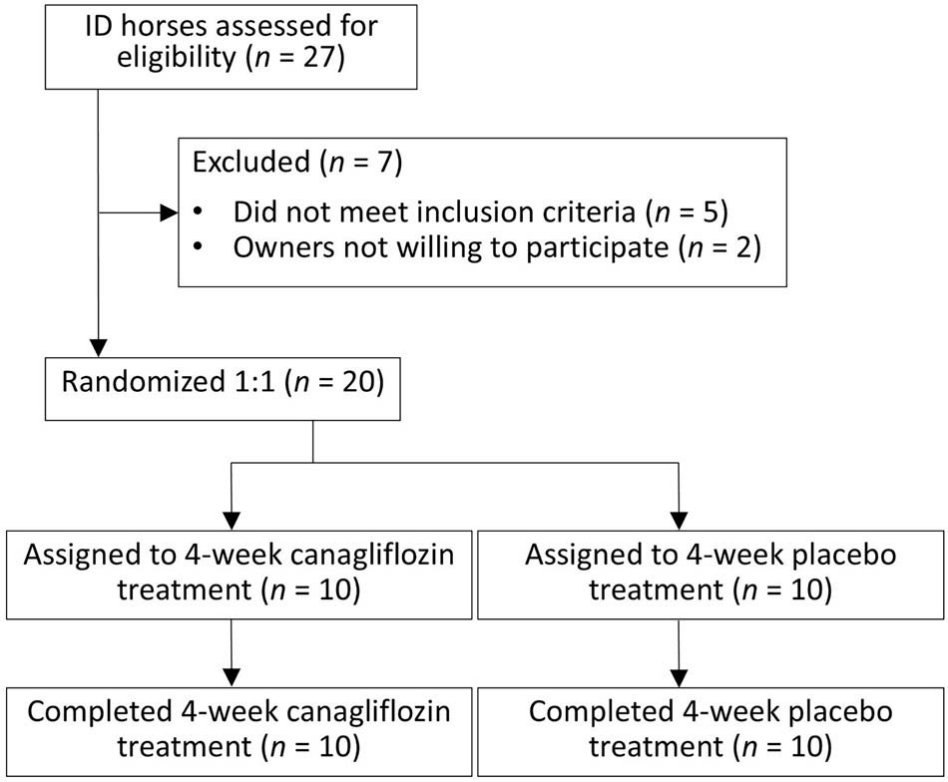
Flow diagram of enrollment. Abbreviation: ID, insulin-dysregulated.

### Experimental protocol

Horses underwent two 4-day examination periods: 1 before and 1 after intervention. During each period, horses were acclimated to the study environment for 2 days. A clinical examination was performed, and body weight, body condition score (BCS),^21^ and cresty neck score (CNS)^22^ were recorded. On day 3, horses underwent a 240-min GGI,^23^^,^^24^ with glucose infused at increasing rates in six 40-min steps (0.4, 0.8, 1.2, 1.6, 2.4, and 3.2 mg/kg/min) to assess β-cell responsiveness to IV glucose. On day 4, a 180-min EHC^25^^,^^26^ was conducted to measure IS and the insulin metabolic clearance rate (MCR). During the post-intervention EHC, an indwelling urinary catheter was used to collect urine for measures of UGE. Blood samples for glucose and insulin were collected throughout. Endogenous insulin concentrations were analyzed using a horse-optimized ELISA,^27^ whereas exogenous insulin during the EHC was measured using a commercial human ELISA.^9^ Plasma and urinary glucose concentrations were measured with an automated clinical chemistry analyzer, and non-esterified fatty acids (NEFA) were quantified using an enzymatic colorimetric assay.^25^

### Outcomes

The primary endpoints were the effect of CAN on IS during EHC, quantified by the glucose disposal rate (M) and the glucose disposal rate per unit of insulin (M/I), with adjustments made for space correction (SC) and UGE. Additionally, glucose disposal rates without correction for UGE (M_uncorrected_ and M/I_uncorrected_) were also included as primary endpoints.

Secondary endpoints included indices of β-cell responsiveness to IV glucose and MCR.

### Statistical methods

Primary and secondary endpoints were analyzed using a linear mixed-effects model, with corresponding baseline (pre-intervention) values included as covariates. Multiple comparisons were adjusted using the Tukey–Kramer post hoc test. Statistical significance was set at *P* < .05. Analyses were performed using JMP Pro 19.0.3.

## Results

### Horses (participants)

Seven horses were excluded before randomization: 5 did not meet the inclusion criteria, and in 2 cases, the owners declined to participate (Figure 1). Of the 20 horses randomized to receive PBO (*n* = 10) or CAN 0.4 mg/kg (*n* = 10), all completed the study. The study sample comprised Icelandic horses (*n* = 7), Shetland ponies (*n* = 7), Welsh ponies (*n* = 2), Spanish native breeds (*n* = 2), a Gotland pony (*n* = 1), and a Haflinger (*n* = 1). Baseline characteristics of the participants are shown in Table 1. No horses exhibited adverse effects related to medication or diagnostic procedures.

**Table 1:** Baseline characteristics of participants.

Variable	Placebo (*n* = 10)	Canagliflozin 0.4 (*n* = 10)
**Age (years)**	14.0 ± 4.3	9.8 ± 4.2
**Body weight (kg)**	339.6 ± 154.9	306.8 ± 121.1
**Breeds**	Shetland ponies (*n* = 4) Icelandic horses (*n* = 3) Spanish breeds (*n* = 2) Gotland pony (*n* = 1)	Shetland ponies (*n* = 3) Icelandic horses (*n* = 4) Welsh ponies (*n* = 2) Haflinger (*n* = 1)
**BCS**	7.5 (6–8)	7 (6–8)
**CNS**	3.5 (3–4)	3 (3–4)
**Previous laminitis (yes/no)**	7/3	8/2
**FPG (mmol/L)**	5.6 ± 0.3	5.5 ± 0.7
**FPI (μIU/mL)**	31.0 (21.1–37.7)	26.3 (20.7–45.3)
**NEFA (mmol/L)**	0.33 (0.30–0.42)	0.36 (0.25–0.58)
**Slope ΔI vs ΔG (mIU/mmol) during graded glucose infusion test**	35.8 (24.4–54.4)	35.8 (18.1–52.6)
**M (mg/kg/min) during euglycemic hyperinsulinemic clamp**	1.5 (1.1–2.0)	1.5 (1.1–2.0)

Values are expressed as *n/n* (count number), mean ± SD or median (interquartile range). Abbreviations: BCS = body condition score; CNS = cresty neck score; FPG = plasma glucose concentration after feed withholding; FPI = plasma insulin concentration after feed withholding; NEFA = non-esterified fatty acid concentration after feed withholding; slope ΔI vs ΔG = slope of the linear regression line of insulin vs glucose concentrations; M = glucose disposal rate.

### Responses to EHC

Calculations were performed using the 100-160-min interval of the EHC. During this period, steady-state conditions were confirmed in all instances, exhibiting low intra-individual variability with a coefficient of variation (CV) < 5% for plasma glucose concentrations (mean CV ± SD: 2.2 ± 0.8%). The mean (± SD) plasma glucose concentration was maintained at 5.0 ± 0.3 mmol/L. Variables related to the EHC, including LS mean differences or ratios and associated *P*-values, are presented in Table 2. The median steady-state glucagon concentrations were <2.0 (<2.0 to 2.4) and <2.0 (<2.0 to <2.0) pmol/L in the PBO and CAN groups, respectively. Data were missing for 6 of 10 PBO-treated horses at steady-state. Glucagon data during the EHC are presented in Table 3.

**Table 2:** Comparison of variables during the euglycemic hyperinsulinemic clamp between placebo- and canagliflozin-treated insulin-dysregulated horses.

Variable	Placebo (*n* = 10)	Canagliflozin (*n* = 10)	Least squares mean difference canagliflozin vs placebo	Geometric least squares mean ratios canagliflozin/placebo (%)	*P*-value
**M (mg/kg/min)**	1.6 ± 0.1	0.7 ± 0.1	−0.9 (-1.3 to -0.5)	n/a	<.001
**M/I (mg/kg/min × 10^3^)/(μIU/mL)**	2.7 ± 0.2	1.5 ± 0.2	−1.3 (-1.9 to -0.6)	n/a	<.001
**M_uncorrected_ (mg/kg/min)**	1.6 ± 0.1	1.4 ± 0.1	−0.2 (-0.6 to 0.1)	n/a	.19
**M/I_uncorrected_ (mg/kg/min × 10^3^)/(μIU/mL)**	2.7 ± 0.3	2.9 ± 0.3	0.2 (-0.6 to 0.9)	n/a	.61
**Mean insulin_steady state_ (μIU/mL)**	592.7 (544.5-645.2)	496.2 (455.9-540.2)	n/a	83.7 (74.2-94.5)	<.001
**MCR (ml/kg/min)**	5.4 ± 0.3	6.5 ± 0.3	1.0 (0.3 to 1.8)	n/a	.01

Note: Means adjusted for baseline values are presented as least squares (LS) means ± SEM or geometric LS means with 95% CI. Least squares mean differences or geometric LS mean ratios for canagliflozin vs placebo are reported with 95% CI. Abbreviations: M = glucose disposal rate corrected for urinary glucose excretion (UGE); M/I = glucose disposal rate per unit of insulin corrected for UGE; M_uncorrected_ = M uncorrected for UGE; M/I_uncorrected_ = M/I uncorrected for UGE; MCR = insulin metabolic clearance rate.

**Table 3:** Glucagon concentrations during the euglycemic hyperinsulinemic clamp in insulin-dysregulated horses after 4 weeks of treatment with canagliflozin (0.4 mg/kg) or placebo.

Variable	Placebo	Canagliflozin
**Glucagon after feed withholding (pmol/L)**	8.4 (5.1-13.1) (*n* = 10)	10.3 (7.1-13.5) (*n* = 10)
**Glucagon at steady state (pmol/L)**	<2.0 (<2.0-2.4) (*n* = 4)	<2.0 (<2.0-2.0) (*n* = 10)

Values are median (interquartile range). Values below 2.0 pmol/L, the lower limit of the assay range, are reported as <2.0 pmol/L

There were no differences between treatments in M or M/I when values were uncorrected for UGE (*P* ≥ .19). After correction for UGE, both M and M/I were lower after CAN treatment compared with PBO (*P* < .001), indicating reduced IS. Least squares mean MCR was higher with CAN treatment (*P* = .01).

### Variables derived from the GGI

Glucose and insulin variables during the GGI are presented in Table 4. Least squares mean plasma glucose concentration after feed withholding (FPG) was 7.3% lower with CAN treatment than with PBO (*P* = .04). The LS mean maximal glucose concentration (glucose C_max_) and LS mean area under the curve for glucose (AUC_glucose_) during the GGI were 15.7% (*P* < .001) and 14.1% (*P* < .001) lower with CAN treatment than with PBO, respectively.

**Table 4:** Comparison of variables during the graded glucose infusion test between placebo- and canagliflozin-treated insulin-dysregulated horses.

Variable	Placebo (*n* = 10)	Canagliflozin (*n* = 10)	Least squares mean differences canagliflozin vs placebo	Geometric least squares mean ratios canagliflozin/placebo (%)	*P*-value
**β-cell responsiveness**					
**Slope ΔI vs ΔG (mIU/mmol)**	36.7 (29.9-45.1)	20.8 (16.9-25.5)	n/a	56.6 (42.3-75.8)	<.001
**iAUC_insulin_/iAUC_glucose_ (mIU/mmol)**	37.4 (30.4-46.1)	19.9 (16.2-24.5)	n/a	53.2 (39.6-71.5)	<.001
**Baseline and response values**					
**FPI (μIU/mL)**	25.9 (19.9-33.7)	17.9 (13.7-23.3)	n/a	69.1 (47.6-100.3)	.05
**Insulin C_max_ (μIU/mL)**	268.0 (215.5-333.3)	123.6 (99.4-153.7)	n/a	46.1 (33.9-62.8)	<.001
**AUC_insulin_ (μIU/mL *×* min)**	28 921 (23 774-35 183)	13 552 (11 140–16 486)	n/a	46.9 (35.5-61.9)	<.001
**FPG (mmol/L)**	5.5 ± 0.1	5.1 ± 0.1	−0.4 (−0.7 to −0.01)	n/a	.04
**Glucose C_max_ (mmol/L)**	12.1 ± 0.3	10.2 ± 0.3	−1.9 (−2.8 to -1.0)	n/a	<.001
**AUC_glucose_ (mmol/L *×* min)**	1 907 ± 39.2	1 639 ± 39.2	−268 (−385 to −151)	n/a	<.001

Means adjusted for baseline values are presented as least squares (LS) means ± SEM or geometric LS means with 95% CI. Least squares mean differences or geometric LS mean ratios for canagliflozin vs placebo are reported with 95% CI. Abbreviations: slope ΔI vs ΔG = slope of the linear regression line of insulin vs glucose concentrations; iAUC_insulin_/iAUC_glucose_ = ratio of incremental area under the curve for insulin and incremental area under the curve for glucose; n/a = not applicable; FPI = plasma insulin concentration after feed withholding insulin; C_max_ = maximal insulin concentration; AUC_insulin_ = area under the curve for insulin; FPG = plasma glucose concentration after feed withholding; glucose C_max_ = maximal glucose concentration; AUC_glucose_ = area under the curve for glucose.

There was no significant difference in plasma insulin concentration after feed withholding (FPI) between groups after treatment (*P* = .05). Geometric LS mean maximal insulin concentration (insulin C_max_) was 53.9% lower (*P* < .001), and area under the curve for insulin (AUC_insulin_) was 53.1% lower (*P* < .001) in the CAN-treated group than in the PBO group during the GGI.

Beta-cell responsiveness during the GGI was lower with CAN treatment compared with PBO (*P* < .001; Figure 2). The geometric LS mean slope of ΔI vs ΔG was 43.3% lower, and the iAUC_insulin_/iAUC_glucose_ ratio was 46.8% lower (*P* < .001) in the CAN-treated group compared with the PBO group. Data for β-cell responsiveness during the GGI are presented in Table 4.

**Figure 2:**
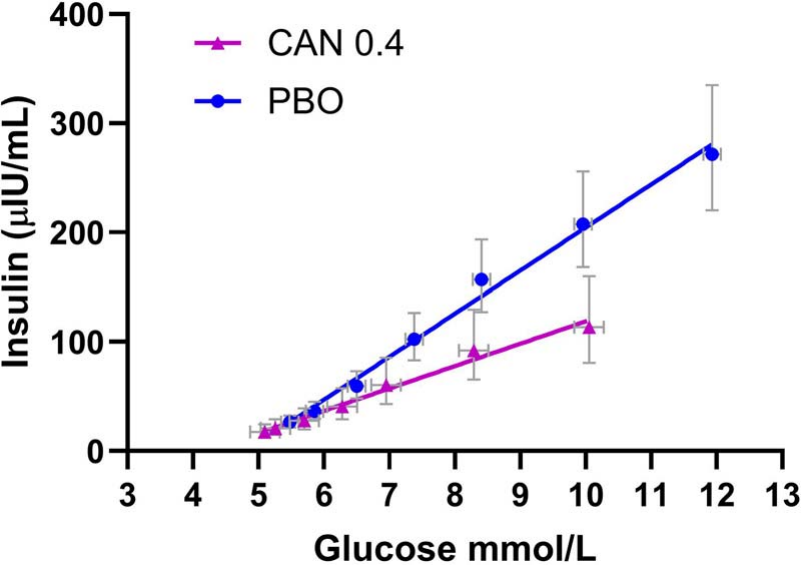
Dose–response relationship between least squares (LS) means (± SEM) glucose and geometric LS means (95% CI) insulin concentrations during the graded glucose infusion test (GGI) in insulin-dysregulated horses receiving 0.4 mg/kg canagliflozin (CAN, triangle), or placebo (PBO, circle). Data were derived from a linear mixed-effects model using plasma glucose and insulin concentrations at 0, 40, 80, 120, 160, 200, and 240 min. The adjusted mean pairs (insulin vs glucose) for each treatment follow a straight line.

### Relationship between **β**-cell responsiveness and IS

Plots illustrating β-cell responsiveness in relation to IS before and after treatment with CAN or PBO are presented in Figure 3. After correction of M/I for UGE, the post-intervention plots for CAN-treated horses show a leftward and downward shift (Figure 3B). In contrast, when β-cell responsiveness was plotted against IS expressed as M/I_uncorrected_, this shift was not observed (Figure 3C).

**Figure 3:**
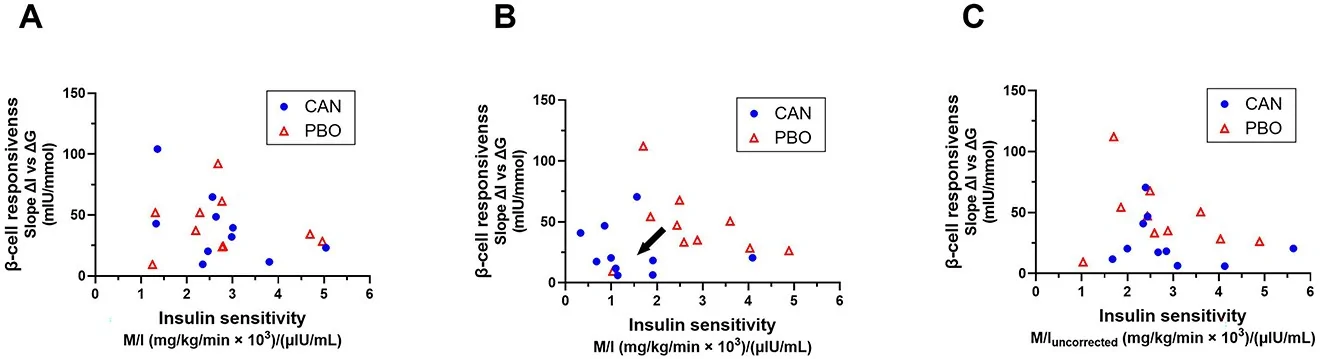
Plot diagram with least squares (LS) mean glucose disposal rate per unit of insulin (M/I) during a euglycemic hyperinsulinemic clamp on the x-axis and β-cell responsiveness on the y-axis. Βeta-cell responsiveness is expressed as the geometric LS mean slope of the linear regression line of insulin vs glucose concentrations (slope ΔI vs ΔG) during the graded glucose infusion test. A: Before treatment, B: After treatment, with M/I corrected for urinary glucose excretion (UGE), C: After treatment, with M/I uncorrected for UGE (M/I_uncorrected_). Circles represent subjects treated with canagliflozin (CAN, 0.4 mg/kg), and triangles represent placebo-treated controls (PBO). In panel B, the disposition index in CAN-treated horses is shifted leftward and downward relative to PBO-treated horses (arrow).

### Urinary glucose concentration and NEFA

Urinary glucose concentrations after feed withholding were higher in the CAN-treated group than in the PBO group (*P* < .001), with a median glucose concentration of 331.3 mmol/L (interquartile range [IQR], 302.7-457.3) vs 0.5 mmol/L (IQR, 0.4-0.6). Assuming constant urine production and UGE over a 24-hour period, the median glucose loss in CAN-treated horses in our study was estimated at approximately 0.85 g/kg body weight per day (IQR 0.76-1.21).

Non-esterified fatty acid concentrations after feed withholding did not differ between CAN- and PBO-treated horses (geometric LS means with 95% CI, 0.50 [0.37-0.68] and 0.33 [0.25-0.45] mmol/L; *P* = .06). However, 1 horse in the CAN group exhibited a clinically deviant pattern, with a markedly lower NEFA concentration after intervention compared with baseline. Exclusion of this outlier revealed that the geometric LS mean NEFA concentration in the CAN group (0.57 mmol/L; 95% CI: 0.45-0.71) was higher (*P* = .002) than in the PBO group (0.33 mmol/L; 95% CI: 0.27-0.41). After treatment, NEFA concentrations were not correlated with IS, as measured by M/I (R^2^ = 0.10; *P* = .18).

### Body weight, BCS and CNS

Body weight was not affected by CAN treatment. Least squares mean ± standard error of the mean (SEM) body weight after the intervention was 315.5 ± 2.0 kg in the CAN group and 319.6 ± 2.0 kg in the PBO group, corresponding to an LS mean difference of -4.1 kg (95% CI, -10.1 to 1.9; *P* = .17). There was also no difference in the percentage change in body weight from baseline to after intervention between the treatment groups (*P* = .27). Body condition score (*P* = .33) and CNS (*P* = .30) did not differ between treatment groups. After intervention, the CAN-treated group had a median BCS of 6 (IQR, 6-8) and a median CNS of 3 (IQR, 3-4), compared with a median BCS of 7 (IQR, 6-8) and a median CNS of 3.5 (IQR, 3-4) in the PBO group.

## Discussion

The primary objective of our study was to determine whether short-term treatment with CAN alters IS in ID horses, as assessed by the EHC, the gold-standard method for evaluating IS. Treatment with CAN was associated with an apparently lower IS. However, further analysis indicated that IS was likely underestimated under the study conditions due to higher glucagon-independent endogenous glucose production (EGP). The observed reduction in measured IS with CAN treatment occurred concurrently with lower insulin concentrations, primarily attributable to lower β-cell responsiveness, with a smaller contribution from higher insulin clearance.

The observation that CAN treatment appeared to lower IS, as determined by M and M/I, was unexpected, as it contrasts with reports in humans showing improved or unchanged IS after treatment with SGLT2 inhibitors^18^^,^^19^^,^^29^ and with a previous study in horses in which IS was unchanged.^17^ In humans with T2D, improvements in IS after SGLT2 inhibitor treatment have been attributed primarily to reductions in plasma glucose concentrations and the associated alleviation of glucotoxicity. In contrast, horses with insulin dysregulation are typically normoglycemic or only mildly hyperglycemic in the unfed state.^30–32^ Consequently, higher IS secondary to reduced glucotoxicity would not be expected in this species, and IS could therefore be expected to remain unchanged.

The M and M/I derived from the EHC quantify glucose uptake in peripheral tissues and rely on the assumption that EGP is fully suppressed to give accurate results. During the EHC, the high insulin infusion rates suppress endogenous insulin and glucagon secretion and typically result in near-complete suppression of hepatic glucose output.^33^^,^^34^ However, if EGP persists during the clamp, the glucose infusion rate required to maintain euglycemia will be lower, leading to an underestimation of M and M/I.

In humans, treatment with SGLT2 inhibitors increases both glucagon concentrations and EGP, with the rise in glucagon at least partly explaining the elevated EGP.^18^^,^^34^^,^^35^ However, even under conditions in which insulin and glucagon secretion are suppressed, such as during clamp conditions, EGP remains higher in humans treated with SGLT2 inhibitors. This indicates the presence of an insulin- and glucagon-independent mechanism stimulating EGP in individuals treated with SGLT2 inhibitors.^34^^,^^36^ In our study, glucagon concentrations measured during the steady-state phase of the EHC were nearly undetectable in all sampled horses, irrespective of treatment, indicating sufficient suppression of glucagon secretion and glucagon-dependent EGP (Table 3). Nevertheless, higher insulin- and glucagon-independent EGP during the EHC in the CAN-treated horses, as observed in humans, is possible. This interpretation is supported by the observation that some CAN-treated horses had calculated M below the proposed insulin-independent glucose disposal rate of approximately 0.57 mg/kg/min^9^, a physiologically implausible result in the absence of concurrent unsuppressed EGP. In humans treated with SGLT2 inhibitors, the increase in EGP is equal or nearly equal to the rate of UGE during fasting or EHC conditions.^18^^,^^37^^,^^38^ If a similar compensatory mechanism occurs in horses, it would explain the selective reduction in calculated M and M/I, while estimates uncorrected for UGE (M_uncorrected_ and M/I_uncorrected_) remain largely unchanged.

The hyperbolic relationship between IS and β-cell responsiveness implies that, when the disposition index, and therefore glucose tolerance, remains constant, lower IS must be accompanied by higher GSIS. In our study, however, GSIS in ID horses was lower with CAN treatment, without a concomitant higher IS. Under normal physiological conditions, this would be expected to result in lower glucose tolerance and progression toward T2D, with elevated blood glucose concentrations.^9^^,^^28^ However, CAN-treated horses exhibited lower, rather than higher, FPG and glycaemic response to IV glucose, and no evidence of T2D was observed, making lower glucose tolerance unlikely. This apparent deviation from the expected hyperbolic relationship cannot be easily explained and further supports the possibility that higher insulin- and glucagon-independent EGP might have led to an underestimation of IS.

Higher concentrations of NEFA have been associated with IR in both humans and horses.^39^^,^^40^ In addition, higher NEFA concentrations have been reported in ID horses treated with ertugliflozin or dapagliflozin.^41^ Collectively, these findings suggest a plausible negative effect of higher NEFA concentration on IS in SGLT2 inhibitor-treated horses. In our study, NEFA concentrations were higher in all CAN-treated horses except one. Thus, a contributory effect of higher NEFA concentrations on the observed lower IS in CAN-treated horses cannot be excluded. However, NEFA concentrations were not correlated with IS as measured by M/I, nor were they associated with M or M/I when included as a covariate in the statistical model. Taken together, these findings suggest that an underestimation of IS is more likely than a true reduction in IS attributable to higher NEFA concentrations.

In our study, β-cell responsiveness during the GGI was lower with CAN treatment than with PBO. The magnitude of this difference was comparable to that reported in a similarly designed study in which horses with insulin dysregulation received higher doses of CAN (0.6 and 1.2 mg/kg).^13^ In that study, the geometric LS mean of the ΔI vs ΔG slope during the GGI was 44.8% and 43.2% lower in the 1.2 mg/kg and 0.6 mg/kg groups, respectively, which is comparable to the 43.3% lower value observed in our study, where the horses received 0.4 mg/kg. Insulin and glucose responses during the GGI, as well as FPG, were lower in CAN-treated horses in our study, whereas FPI showed no clear difference from PBO (*P* = .05). Collectively, these results suggest that short-term treatment with CAN at 0.4 mg/kg resulted in lower insulin concentrations in horses with insulin dysregulation to a degree comparable to that reported with higher doses, at least in response to an IV glucose challenge.

Lower insulin clearance compared with healthy individuals has been suggested in horses with insulin dysregulation, although the evidence is limited.^7^^,^^42^ In a separate study, mean insulin clearance during EHC in sedentary, forage-fed Standardbred mares with normal insulin regulation was 5.07 and 4.90 mL/kg/min before and after weight gain, respectively.^25^ These values are comparable to, and in some cases lower than, the 5.4 mL/kg/min observed in the PBO group in the present study, highlighting heterogeneity in insulin clearance within both healthy and ID populations of horses. In contrast, reduced insulin clearance is more consistently demonstrated in humans with obesity or T2D.^16^^,^^43^^,^^44^ However, the relative contribution of lower insulin clearance to the development of hyperinsulinemia in horses with insulin dysregulation, compared with other mechanisms such as higher β-cell responsiveness and the enhanced incretin effect, remains unclear.^7^^,^^42^ In our study, insulin clearance during the EHC was on average 20.4% higher with CAN treatment than with PBO. Results in other species are somewhat inconsistent, although higher insulin clearance after treatment with SGLT2 inhibitors are reported.^45^^,^^46^ Overall insulin exposure during the GGI, measured as the AUC_insulin_, was on average 53.1% lower with CAN treatment than with PBO. This substantial reduction in circulating insulin concentrations likely reflects the combined effects of lower β-cell responsiveness and higher insulin clearance under treatment with SGLT2 inhibitors. The higher insulin clearance observed here represents a potentially important mechanism contributing to the insulin-lowering effect of CAN, in addition to its previously reported effect on β-cell responsiveness.^4^^,^^13^ These findings suggest that modulation of insulin clearance might be an additional therapeutic pathway through which SGLT2 inhibition mitigates hyperinsulinemia in ID horses. However, as insulin clearance was assessed under experimentally induced hyperinsulinemia rather than under physiological conditions, the extent to which this effect translates into the postprandial state remains to be investigated.

Canagliflozin resulted in higher UGE in all treated horses. The median urinary glucose concentration in CAN-treated horses after feed withholding, 10-12 hours after medication, was 331.3 mmol/L, which is similar to mean concentrations (277-347 mmol/L) reported 24-72 hours after administration of a single dose of CAN (1.8 or 3.6 mg/kg) in healthy horses.^47^ Assuming constant urine production and UGE over a 24-hour period, the median glucose loss in CAN-treated horses in our study was estimated at approximately 0.85 g/kg body weight per day. This magnitude of glucosuria is comparable to the approximately 70 g of glucose lost over 24 hours reported in healthy humans with normal blood glucose concentrations treated with CAN, although participant body weights are not reported.^48^

It is possible that the calculated 24-hour glucose loss in the horses is underestimated, since both UGE and urine production were measured after feed withholding and would likely be higher in the fed state. Regardless, the glucose loss induced by SGLT2 inhibitors is considerable and might cause a substantial energy deficit. Weight loss in ID horses treated with SGLT2 inhibitors is reported in some studies.^4^^,^^49^ However, despite higher UGE and instructions to horse owners to maintain a consistent feeding regimen, no significant weight loss was observed in CAN-treated horses in our study. This could reflect limited statistical power, as the study was not designed to detect changes in body weight, the relatively short study duration, or incomplete control of feed intake.

Our study has some limitations. First, although the EHC is considered the gold standard for measuring IS, its interpretation might be complicated in subjects receiving SGLT2 inhibitors. These drugs can cause higher EGP independently of insulin and glucagon,^35^^,^^36^ which might confound estimation of IS when tracer glucose is not used. Although tracer glucose has been used in horses,^50^ they require specialized equipment and were not used in our study due to practical constraints. Second, insulin clearance was assessed during the steady state of the EHC, a non-physiological condition characterized by markedly elevated insulin concentrations due to peripherally administered exogenous insulin. Because insulin clearance is a dynamic process, values obtained during the EHC might differ from those observed under physiological conditions, such as during the GGI or in the postprandial state, where insulin is endogenously secreted from the pancreas.^51^ Determination of endogenous insulin clearance during the GGI, and evaluation of its contribution to reduced insulin secretion, would have benefited from concurrent analysis of C-peptide. C-peptide is secreted from pancreatic β-cells in equimolar amounts with insulin; however, unlike insulin, which is metabolized by the liver by approximately 50% in healthy humans, C-peptide enters the systemic circulation unmetabolized and is therefore considered a more accurate indicator of β-cell secretion.^52^ At the time this study was conducted, however, validated assays for C-peptide analysis were not available for use in horses, precluding such measurements. Nevertheless, in a study in humans in which β-cell responsiveness was assessed by a GGI, close concordance between insulin and C-peptide responses was observed.^53^ This observation suggests that the results from the GGI in our study likely provide a reasonable estimate of the β-cell responsiveness rather than reflecting changes in insulin clearance, although similar studies have not yet been conducted in horses. Finally, only mares were included in the study. This decision was made because sedative agents required for urinary catheter placement in geldings and stallions might affect glucose and insulin responses.^54^^,^^55^ Although this limits the generalizability of the findings, sex has not been shown to influence insulin metabolism in horses,^32^ making it unlikely that the restriction to mares substantially affected the study outcomes.

In summary, treatment with CAN reduced IS as measured by the EHC; however, concurrent higher glucagon-independent EGP might have led to an underestimation of IS in treated mares. Furthermore, insulin responses to IV glucose were markedly lower with CAN treatment, consistent with reduced β-cell responsiveness. However, higher insulin clearance also contributed to the lower insulin response. Collectively, these findings suggest that CAN reduces hyperinsulinemia in mares with insulin dysregulation through multiple metabolic mechanisms, without concurrently improving IS.

## Off-label antimicrobial declaration

Authors declare no off-label use of antimicrobials.

## Institutional animal care and use committee or other approval declaration

The study protocol was approved by the Ethics Committees in Sweden (Dnr 5.8.18-14166/2023).

## Appendix: Materials and methods

### Study design

This was a single-center, double-blind, parallel-group, placebo-controlled study. Privately owned horse and pony mares (hereafter collectively referred to as “horses”) were randomized in a 1:1 ratio to receive either CAN (0.4 mg/kg) or placebo (PBO) administered PO once daily for 4 weeks. The study was conducted at the equine clinic at the Animal Hospital at the Swedish University of Agricultural Sciences between October 2023 and April 2025. The horses were examined before the start of the treatment to obtain baseline data and were re-examined at the end of the treatment period (post-intervention). Each examination period included 2 days of acclimatization, followed by a GGI on day 3 (corresponding to 27 days of treatment at the revisit) to assess β-cell responsiveness and an EHC on day 4 (corresponding to 28 days of treatment at the revisit) to measure IS and insulin clearance. Written informed consent was obtained from all owners before enrollment. Owners were instructed to maintain the horses’ feeding and training routines unchanged for 1 month before study initiation and throughout the study period.

### Enrollment criteria

Privately owned horses diagnosed with insulin dysregulation based on an OST were eligible for enrollment. The OST was performed by referring veterinarians and consisted of PO administration of glucose syrup (Dansukker, Nordic Sugar; 0.2 mL/kg body weight) followed by blood sampling for insulin measurement 60-90 min post-administration. Horses with insulin concentrations >100 μIU/mL were eligible for inclusion; concentrations >45 μIU/mL are considered diagnostic for ID (Clinical Pathology Laboratory, Swedish University of Agricultural Sciences). Exclusion criteria included horses <2 years of age, geldings and stallions (as urinary catheter placement without sedation is anatomically difficult), pregnant or lactating mares, and horses with signs of ongoing laminitis. Additional exclusion criteria included clinical evidence of pituitary pars intermedia dysfunction, other systemic disease, or current treatment with any therapeutic agents. A flow diagram of enrollment is shown in Figure 1.

### Experimental protocol

Horses were examined for two 4-day periods, pre- and post-intervention, at the equine clinic. A clinical examination was performed, and body weight, BCS,^21^ and CNS^22^ were recorded at both visits. Horses were randomized in a 1:1 ratio using a web-based randomization service (www.randomizer.org) to receive once-daily PO treatment with either CAN 0.4 mg/kg or PBO. The treatment was blinded to owners, the personnel handling the horses, and the investigators conducting the EHC and scoring. To achieve the precise dose, commercial 100 mg or 300 mg CAN tablets (Invokana, Mundipharma AB, Gothenburg, Sweden) were weighed and divided as needed using a scalpel blade. Since identical PBO tablets were unavailable, PBO was prepared by filling vegetable capsules (Tomma Kapslar, Närokällan) with approximately 0.75 g of lactose powder. This powder matched the lactose excipient naturally found in the Invokana tablets. Both Invokana tablets and PBO capsules were dispensed in identical plastic containers, which were placed in a paper box with administration instructions, and handed over to the owners. During the revisit, medication was administered at 9 pm, 1 hour before the last feeding of the day. Between the examination periods, the horses were treated at home by their owners, and the medication was administered between 7 and 10 pm. The tablet or PBO capsule was placed in a small piece of apple and administered by hand at the time of dosing.

During examination periods, horses were housed in box stalls where all procedures were conducted. Horses were provided daily access to a sand paddock. Each examination period began with a 2-day acclimatization phase. Horses were fed their own regular diet, consisting of only forage supplemented with vitamins and minerals, divided into 4 daily feedings. Water was available ad libitum. Feed was withheld for 8 hours overnight before both the GGI and the EHC. Body weight was recorded using an electronic scale before each test.

On day 2, bilateral jugular vein catheters (Intranule, 2.0 × 105 mm, Vygon France or Milacath® − extended use 1.7 × 75 mm, Mila, USA, depending on the size of the horse) were placed aseptically using topical (EMLA, AstraZeneca AB, Södertälje, Sweden) and subcutaneous local anesthesia (Carbocain, 20 mg/ml, Aspen Nordic, Dublin, Ireland or Mepidor vet, 20 mg/mL, Salfarm Scandinavia, Helsingborg, Sweden).

### Graded glucose infusion test

On day 3, starting at approximately 7 am, horses underwent a 240-min GGI using a protocol adapted for equines.^23^^,^^24^ Blood samples for glucose, insulin, and biochemical variables were collected 5 min before initiation of the GGI. An additional blood sample for glucose analysis was obtained 1 min before the start of the glucose infusion (Glucose Fresenius Kabi 200 mg/mL, Fresenius Kabi, Uppsala, Sweden). The glucose infusion was administered via one of the IV catheters at an initial rate of 0.4 mg/kg/min, with the rate increased every 40 min to 0.8, 1.2, 1.6, 2.4, and 3.2 mg/kg/min. Blood samples for glucose analysis were collected every 10 min from the contralateral IV catheter, and blood samples for insulin were obtained at 30 and 40 min after each increase in glucose infusion rate.

Maximum (C_max_) glucose and insulin concentrations were defined as the highest values observed during the GGI. The area under the curve (AUC) for glucose and insulin responses was calculated using the trapezoidal method. Incremental AUC (iAUC) was obtained by subtracting the area corresponding to fasting baseline concentrations (AUC—[fasting concentration *×* 240]).

Two indices of β-cell responsiveness to IV glucose were used, reflecting the ability of β-cells to sense changes in prevailing glucose concentrations and appropriately adjust insulin secretion. The first index (ΔI vs ΔG) was derived from a simple linear regression analysis of the insulin vs glucose concentrations obtained during each GGI.^13^^,^^56^ This analysis included fasting concentrations (time 0 min) and those measured at the end of each glucose infusion step. The slope of this regression (ΔI vs ΔG) was used as the index. The second index was calculated as the ratio of iAUC_insulin_ to iAUC_glucose_ (iAUC_insulin_/iAUC_glucose_).^13^

### Euglycemic hyperinsulinemic clamp

On day 4, starting in the morning, a 180-min EHC was performed as previously described.^25^^,^^26^ Briefly, insulin (Humulin®Regular, 100 IE/mL, Lilly, Solna, Sweden) diluted in saline (Natriumklorid, Fresenius Kabi, Uppsala, Sweden) was administered via one of the IV catheters at a constant rate of 3.0 mU/kg/min. Concurrently, a glucose solution (Glucose Fresenius Kabi, 500 mg/mL, Fresenius Kabi, Uppsala, Sweden) was infused at a variable rate to maintain blood glucose at euglycemic concentrations (5 mmol/L).

Blood samples for glucose analysis were collected from the IV catheter not used for the glucose and insulin infusions at 10 and 1 min before the start of the infusion, and every 5 min throughout the clamp procedure. Blood glucose concentrations were measured immediately using a handheld glucometer (Roche Diagnostics, Scandinavia AB, Bromma, Sweden) to guide adjustment of the glucose infusion rate. The infusion rate was adjusted whenever glucose concentrations deviated by more than 0.2 mmol/L from the target euglycemic concentration. In addition, blood samples for glucose analysis were collected every 10 min during the EHC for subsequent laboratory analyses. Blood samples for insulin analysis were collected at baseline and every 20 min throughout the test. Glucagon concentrations were measured in the fasted state and at steady state (140-160 min after start of the clamp) during the EHC. Values below 2.0 pmol/L, the lower limit of the assay range, were reported as <2.0 pmol/L.

During the second examination period, an indwelling urinary catheter (C.R. Bard, Inc., Covington, United States) was placed after aseptic preparation. Anesthetic gel (Xylocain 2%, Aspen Nordic, Dublin, Ireland) was applied to the catheter before insertion. The urinary bladder was emptied using a syringe immediately before the start of the clamp. Urine samples were collected, transferred to plastic tubes, and centrifuged within 5 min, and supernatant was collected. Thereafter, the catheter was connected to a collection bag (Bed Bag, 2000 mL, Qufora, Tianchang City, China). At the end of the examination, urine was transferred from the collecting bag to a graduated cylinder, where the volume was recorded, and additional urine samples were collected, transferred to plastic tubes, and centrifuged. After completion of the clamp, the insulin infusion was stopped, and the horses were fed forage and a carbohydrate-rich concentrate while blood glucose concentrations were monitored. Once blood glucose concentrations stabilized above 5 mmol/L, the glucose infusion was discontinued. The IV catheter was removed if no hypoglycemic events occurred within 30 min after completion of the infusion.

The overall most stable 60-min period during the EHC for all horses was identified by comparing the average CV of plasma glucose concentrations within the steady-state interval (90-180 min). The period with the lowest CV% was selected and used for all subsequent calculations of IS and insulin clearance.

The glucose disposal rate (M) was calculated as the average steady-state glucose infusion rate (GIR), corrected by subtracting the rate of SC and the estimated rate of UGE for the 60-min period^57^:

$$\mathrm{M}\ \left(\mathrm{mg}/\mathrm{kg}/\mathrm{min} \right)=\mathrm{GIR}\ \left(\mathrm{mg}/\mathrm{kg}/\mathrm{min} \right)-\mathrm{SC}-\mathrm{UGE}$$

The SC (mg/kg/min) represents a calculation that accounts for glucose entering or leaving the glucose distribution space. A glucose distribution volume of 0.19 L/kg^58^ was assumed for this calculation:

$$\mathrm{SC}\ \left(\mathrm{mg}/\mathrm{kg}/\mathrm{min} \right)=\left({\mathrm{G}}_1-{\mathrm{G}}_0\right)\times 0.19/\mathrm{time}\ \mathrm{period}\ \left(\mathrm{min} \right)$$

where G_1_ and G_0_ represent plasma glucose concentrations at the end and the beginning of the time period, respectively. Because individuals with varying degrees of ID exhibit substantial differences in mean insulin concentrations during the EHC, the glucose disposal rate (M) was normalized to mean steady-state insulin concentrations (M/I). In addition, uncorrected indices of glucose disposal were calculated without adjustment for UGE (M_uncorrected_ and M/I_uncorrected_). The EHC was considered stable if the CV% of plasma glucose concentrations during the selected steady-state period was <5% for each individual run.

The clearance rate of insulin was expressed as the MCR and calculated as:

\begin{align*}& \mathrm{MCR}\ \left(\mathrm{ml}/\mathrm{kg}/\mathrm{min} \right)=\mathrm{insulin}\ \mathrm{infusion}\ \mathrm{rate}/\left(\mathrm{steady} \mathrm{-} \mathrm{state}\right.\nonumber\\&\quad\left.\ \mathrm{mean}\ \mathrm{insulin}\ \mathrm{concentration} - \mathrm{fasting}\ \mathrm{plasma}\ \mathrm{insulin}\right.\nonumber\\&\quad \left.\ \mathrm{concentration}\ \left[\mathrm{FPI}\right]\right) \end{align*}

### Sample collection

All blood samples were collected into tubes containing lithium heparin for glucose and insulin analysis, EDTA for glucagon analysis, and into tubes without additives for the analysis of NEFA concentrations. Lithium heparin tubes were centrifuged within 5 min of collection, whereas tubes without additives were allowed to clot before centrifugation. After centrifugation, plasma and serum were harvested from blood samples. All samples, including plasma, serum, and urine, were immediately frozen at -80 °C. Endogenous insulin concentrations during the GGI were analyzed in duplicate using a horse-optimized ELISA (Mercodia Equine Insulin ELISA; Mercodia AB, Uppsala, Sweden)^27^, whereas exogenous insulin (human recombinant insulin) during the EHC was measured using a commercial human ELISA (Insulin ELISA; Mercodia AB, Uppsala, Sweden).^9^ Plasma glucose concentrations and urinary glucose concentrations were analyzed using an automated clinical chemistry analyzer (DxC 700 AU from Beckman Coulter) previously used in equine research.^41^^,^^47^ Serum NEFA concentrations were measured using a commercially available enzymatic colorimetric assay (NEFA-HR (2), ACS-ACOD Method, Wako Chemicals GmbH, Neuss, Germany) applicable to veterinary samples and previously used in equine research.^25^ Glucagon concentrations were measured using a commercially available ELISA validated for use in horses (Glucagon ELISA, Mercodia AB, Uppsala, Sweden).^58^

### Outcomes

The primary endpoint was the difference in IS between horses treated with CAN 0.4 mg/kg and PBO, assessed using the EHC. Insulin sensitivity was quantified using the M and the M/I. Secondary endpoints included insulin MCR, determined during the EHC, and β-cell responsiveness, as well as insulin and glucose responses during the GGI. Additional secondary endpoints included fasting and maximal glucose and insulin concentrations during the GGI, NEFA concentrations, and body weight. β-cell responsiveness was assessed by the slope of the linear regression line of insulin vs glucose concentrations (ΔI vs ΔG) and by the ratio of the incremental areas under the curve for insulin and glucose (iAUC_insulin_/iAUC_glucose_). Insulin and glucose responses were determined by the AUC and by the iAUC during the GGI.

### Calculations and statistical methods

All statistical analyses were performed using JMP Pro version 19.0.3 (SAS Institute Inc, Cary, NC).

A sample size of 10 horses per treatment group was estimated to provide >80% power to detect a change in M (mean ± SD) from 1.4 ± 0.3 to 1.8 ± 0.3 mg/kg/min, using a two-sided *t-*test with an α level of 0.05. It was assumed that ID horses in this study would have a baseline mean M of 1.4 mg/kg/min. The expected change in M was based on previous research indicating that β-cell responsiveness decreases by more than 40% after CAN treatment.^4^^,^^13^ Given the hyperbolic relationship between β-cell responsiveness and IS, such a decrease in β-cell responsiveness is expected to correspond to an increase in mean M from 1.4 to 1.8 mg/kg/min.^9^

A linear mixed-effects model was used to analyze primary and secondary endpoints with treatment included as a fixed effect. The corresponding baseline values (pre-intervention data) were included as a continuous covariate to adjust for baseline differences. The full model included an interaction between treatment and baseline values; however, this interaction was not significant and was therefore excluded from the final analyses.

Visualization of the overall dose–response relationship between treatments for insulin vs glucose concentrations used LS means ± SEM for glucose and geometric LS means (95% CI) for insulin from a mixed-effects model. Glucose and insulin responses over time at 0, 40, 80, 120, 160, 200, and 240 min during the GGI after treatment were analyzed using mixed-effects models for each treatment group, with sampling time point as fixed effect and pre-treatment AUC_glucose_ or log-transformed AUC_insulin_ as continuous covariates.

Residual plots and Q-Q plots were used to assess homoscedasticity and normality of residuals, respectively. Data were log-transformed when either assumption was violated. Adjusted means for normally distributed data were presented as LS mean ± (SEM). Data that were log-distributed were reported as geometric LS means and 95% CI after back transformation to the original scale. For multiple comparisons between treatments, the Tukey–Kramer post hoc test was used. Ordinal data were analyzed using a Wilcoxon two-sample test. Values with *P* < .05 were considered statistically significant.

## Abbreviations

AUC_glucose_: area under the curve for glucose
AUC_insulin_: area under the curve for insulin
BCS: body condition score
CNS: cresty neck score
CV: coefficient of variation
EGP: endogenous glucose production
EHC: euglycemic hyperinsulinemic clamp
FPG: plasma glucose concentration after feed withholding
FPI: plasma insulin concentration after feed withholding
GGI: graded glucose infusion test
Glucose C_max_: maximal glucose concentration
GSIS: glucose-stimulated insulin secretion
HOMA-IR: homeostasis model assessment of insulin resistance
iAUC_glucose_: incremental area under the curve for glucose
iAUC_insulin_: incremental area under the curve for insulin
iAUC_insulin_/iAUC_glucose_: ratio of incremental area under the curve for insulin and glucose
ID: insulin-dysregulated
Insulin C_max_: maximal insulin concentration
IQR: interquartile range
LS mean: least squares mean
M: glucose disposal rate corrected for urinary glucose excretion
M/I: M per unit of insulin
M_uncorrected_: M uncorrected for urinary glucose excretion
M/I_uncorrected_: M_uncorrected_ per unit of insulin
MCR: insulin metabolic clearance rate
NEFA: non-esterified fatty acids
OST: oral sugar test
SC: space correction
SGLT2: sodium-glucose co-transporter 2
SEM: standard error of the mean
T2D: type 2 diabetes
UGE: urinary glucose excretion
ΔI vs ΔG: slope of the linear regression line of insulin vs glucose concentrations
